# Insights into the Genome of the Anaerobic Acetogen *Sporomusa silvacetica* DSM 10669

**DOI:** 10.1128/genomeA.00983-17

**Published:** 2017-09-21

**Authors:** Jonathan R. Humphreys, Rolf Daniel, Anja Poehlein

**Affiliations:** a Clostridia Research Group, BBSRC/EPSRC Synthetic Biology Research Centre (SBRC), School of Life Sciences, University of Nottingham, Nottingham, United Kingdom; b Genomic and Applied Microbiology & Göttingen Genomics Laboratory, Georg-August University Göttingen, Göttingen, Germany

## Abstract

*Sporomusa silvacetica* is a spore-forming, anaerobic acetogen isolated from soil derived from east central Germany. The genome contains genes of the Wood-Ljungdahl pathway required for carbon fixation and genes involved in the biosynthesis of the amino acid pyrrolysine. The genome (5.92 Mb) harbors 4,355 predicted protein-encoding genes.

## GENOME ANNOUNCEMENT

*Negativicutes*, a class within the phylum *Firmicutes*, contains the anaerobic, banana-shaped, endospore-forming acetogens belonging to the genus *Sporomusa*. Species within this genus are able to metabolize a variety of substrates including methanol, ethanol, betaine, and *N*,*N*-dimethylglycine (1). As *Sporomusa* strains are able to catalyze the formation of acetate from H_2_-CO_2_ using the Wood-Ljungdahl pathway, they are potential industrial candidates for waste gas usage (1–3). *Sporomusa silvacetica* DSM 10669 was isolated from aggregate forest soil derived from east central Germany, an area that is often subject to changes in aeration and redox potential (4). The adaptability of some *Sporomusa* strains to environments that are not strictly anaerobic is of interest, as these strains may be more tolerant to industrial fluctuations. Here, we present the draft genome of *S. silvacetica* DSM 10669.

Chromosomal DNA of *S. silvacetica* DSM 10669 was isolated using the MasterPure complete DNA purification kit (Epicentre, Madison, WI, USA). The extracted DNA was used to generate Illumina shotgun paired-end sequencing libraries, which were sequenced with a MiSeq instrument and the MiSeq reagent kit version 3 as recommended by the manufacturer (Illumina, San Diego, CA, USA). Quality filtering using Trimmomatic version 0.32 (5) resulted in 3,234,427 paired-end reads. The assembly performed with the SPAdes genome assembler software version 3.9.0 (6) resulted in 165 contigs (>500 bp) with an average coverage of 107-fold. The assembly was validated and the read coverage determined with QualiMap version 2.1 (7). The draft genome of *S. silvacetica* DSM 10669 consisted of a single chromosome (5,929,109 bp), with an overall G+C content of 42.95%. Automatic gene prediction and identification of rRNA and tRNA genes were performed using the software tool Prokka (8). The draft genome contained 14 rRNA genes, 93 tRNA genes, 4,355 protein-encoding genes with predicted functions, and 1,285 genes coding for hypothetical proteins.

The gene cluster encoding the enzymes of the Wood-Ljungdahl pathway for carbon fixation was present in the genome and showed the same orientation as the ones of *S. ovata* (9) and *S. sphaeroides* (10). Genes encoding the enzymes PylB, PylC, and PlyC involved in the biosynthesis of the amino acid pyrrolysine were also found. Pyrrolysine is known as the 22nd amino acid and is encoded by the recoding of the stop codon UAG (11). Genes encoding the Rnf complex and several gene cluster coding for hydrogenases were also present.

### Accession number(s).

This whole-genome shotgun project has been deposited at DDBJ/EMBL/GenBank under the accession no. LSLK00000000. The version described here is the first version, LSLK01000000.
